# Evaluation of drug lag and drug loss in Japan: participation in global phase III oncology trials

**DOI:** 10.1007/s10147-025-02756-8

**Published:** 2025-04-11

**Authors:** Kaname Shiga, Taro Shibata, Toshio Miyata

**Affiliations:** 1 Cooperative Major in Advanced Biomedical Sciences, Joint Graduate School of Tokyo Women’S Medical University and Waseda University, Waseda University, 2-2 Wakamatsucho, Shinjuku, Tokyo, 162-8480 Japan; 2 Biostatistics Division, Center for Research Administration and Support, National Cancer Center, Tokyo, Japan; 3 Medical Affairs Division, Johnson & Johnson, Tokyo, Japan

**Keywords:** Drug lag, Drug loss, Anticancer drug development, MRCT, Phase III

## Abstract

**Background:**

Despite efforts to mitigate drug lag, discrepancies in drug approval timelines persist between Japan and the US, and increase in unapproved drugs has become a significant challenge. This study aimed to evaluate potential drug lag and drug loss by assessing Japan’s participation in global phase III multinational/multiregional clinical trials (MRCTs) targeted cancers.

**Methods:**

Phase III MRCTs of anticancer drugs initiated between 2008 and 2022 were collected. Information of participant countries, study sponsor, study design, and cancer type were collected and analyzed by logistic regression analysis to identify factors affected Japan’s participation.

**Results:**

Of 999 phase III MRCTs, Japan’s participation every 5 years increased over 15 years (2008–2012: 34.3%, 2013–2017: 51.6%, 2018–2022: 60.2%), while Japan’s non-participation numbers did not change (2008–2012: 157, 2013–2017: 167, 2018–2022: 165). In the multivariate logistic regression analysis, the absence of an operational base in Japan and minor cancers were negatively associated with Japan’s participation in phase III MRCTs. Japan’s participation was also associated with some cancer organs and drug modalities.

**Conclusion:**

Potential future drug lag and increases of unapproved drugs were expected to increase. Since the inclusion of Japan in MRCTs results in shorter or no approval lag, Japan should promote to make circumstances where small overseas companies can include Japan in MRCTs.

**Supplementary Information:**

The online version contains supplementary material available at 10.1007/s10147-025-02756-8.

## Introduction

Cancer is a serious and fatal disease, accounting for approximately 25% of all deaths in Japan [1], and it is reported that 65.5% of Japanese men and 51.2% of women will be diagnosed with cancer during their lifetime [2]. As a result, there is a need for new and innovative drugs to improve the prognosis of cancer patients, and clinical development of anticancer drugs is active in Japan and overseas.

However, there is a difference in the timing of anticancer drug approvals between Japan and other countries, particularly the US. This problem is known as “drug lag” and is a serious issue because it affects patient access to medicines, especially anticancer drugs [3, 4].

From the late 2000s to the early 2010s, the Ministry of Health, Labour and Welfare (MHLW) issued several guidelines and notifications to promote the use of multinational/multiregional clinical trials (MRCTs) to support the marketing authorization of new drugs in Japan [5–7].

In recent years, the development of clinical trial systems and expansion of capabilities of the Pharmaceuticals and Medical Devices Agency (PMDA) system have narrowed the gap between Japan and the US in the development of anticancer drugs [8, 9], but the issue of “drug loss”, where drugs are not developed since there are no companies to develop them in Japan and the number of unapproved drugs increases in the future, has become a major challenge [10, 11].

Previous reports have shown that Japanese participation in MRCTs is associated with both drug submission and approval lags [12, 13]. It has been reported that the approval lag is shorter when Japan is included in an MRCT than when a separate local trial is conducted in Japan [14]. Therefore, including Japan in MRCTs is advantageous for pharmaceutical companies as it makes it easier to obtain approval in the Japanese market. In addition, recent reports indicate that the potential for future approval lags and undeveloped drugs can be measured by the rate of Japanese participation in MRCTs. It has been reported that 80% of drugs not participating in MRCTs for anticancer, anti-infective, antipsychotic, and cardiovascular drugs were not developed in Japan (potential drug loss) [15].

Investigating the reasons why it is difficult to include Japan in MRCTs will also contribute to efficient drug development strategies for drugs of foreign origin.

This study, therefore, aimed to investigate the situation of “potential drug lag/drug loss” based on Japan’s participation rate in phase III MRCTs of anticancer drugs, explored associated factors.

## Materials and methods

### Criteria for trial selection

This study included phase III MRCTs that were funded by pharmaceutical companies, included the US as one of the participant countries, and targeted cancer (either solid tumors or hematological malignancies), started between 01/01/2008 and 12/31/2022. Trials that did not intend efficacy to cancers, conducted only in the US, and were not led by pharm, extension, or rollover studies were excluded.

### Data sources and collected information

Data were collected from ClinicalTrials.gov (https://clinicaltrials.gov/). The collected information includes study start year, participant country, sponsor, type of cancer, study drug, study design, sample size, and primary endpoint.

### Cancer incidence

To investigate the difference in Japan’s participation by cancer incidence, the cancer targeted by each clinical trial was categorized by incidence as either major (6 or more cases/100,000 individuals/year) or minor (< 6/100,000 individuals/year) by utilizing the definition for rare cancer by International Rare Cancers Initiative (https://project.eortc.org/irci/) (RARECARE definition) [16–18].

### Cancer organ

Each cancer was classified to each organ (biliary tract, brain, breast, cervix, colorectum, esophagus, head and neck, kidney, liver, lung, mesothelium, neuroendocrine, ovary, pancreas, prostate, skin, soft tissue, stomach, thyroid, urothelium, and uterus). Hematologic malignancies were classified as leukemia, lymphoma, and myeloma [19].

### Drug modality

Each drug modality was classified according to the existing report as antibodies (including monoclonal antibodies, antibody–drug conjugates, and bispecific antibodies), protein and peptides, cell therapies (including CAR-T), gene therapies, nucleic acids (DNA and RNA), other new modalities (including oncolytic viruses), small molecules (cytotoxic agents and molecular targeted drugs), and radiotherapies (including radio-drug conjugates) [20].

### Company size definition

To investigate the relevance of company size in Japan’s participation, each company was categorized as either “Large company” or “Non-large company” based on global sales ranking top 20 between 2018–2022. If the company was involved in top 20 multiple times, the company was categorized as “Large company”. The Large companies are following: Pfizer, Johnson&Johnson (Janssen), Roche/Genentech, Merck (MSD), AbbVie (Abbott), Novartis, Bristol Myers Squibb, Sanofi, AstraZeneca, Glaxosmithkline (GSK), Takeda, Eli Lilly, Bayer, Amgen, Gilead Sciences, Boehringer Ingelheim. Subsidiaries of large companies were classified as “Large company”.

### Outcome measures and statistical analyses

The participation rate of the target countries was calculated as the number of target countries included in the total number of phase III MRCTs. The US participation rate was assumed to be 100%. The five most populous European countries (Germany, UK, France, Italy, and Spain) were grouped together for analysis as the “EU5”. East Asia countries (Japan, Korea, China, Hong Kong, and Taiwan) were included in the “East Asia Total”. Also, the participation number and its difference between the US and Japan, and between East Asia total and Japan were calculated to investigate Japan’s non-inclusion to phase III MRCTs.

Univariate and multivariate logistic regression analyses were performed using the extracted trial characteristics to explore factors affecting Japanese participation/non-participation in oncology phase III MRCTs. Odd ratios and 95% confidence intervals were calculated. For univariate analysis, the following factors were analyzed: company size (large/non-large), operational base in Japan, number of countries, sample size, study start year, study design (comparative/non-comparative), primary endpoint (overall survival/surrogate), cancer incidence (major/minor), and cancer type (solid tumors/hematological malignancies). For multivariate analysis, the following independent variables were used: operational base in Japan, number of countries, study start year, primary endpoint, and cancer incidence.

Statistical analyses were performed using JMP®Pro and statistical significance was set at *p* < 0.05.

## Results

### Clinical trials selected

**Fig. 1:**
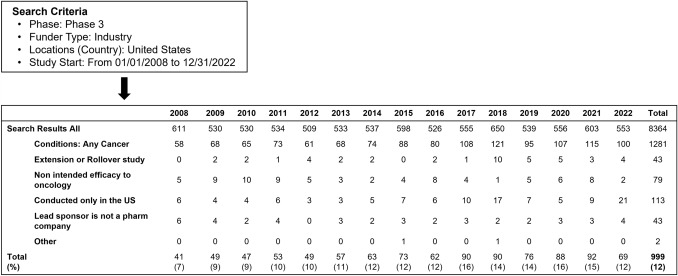
Phase III multiregional clinical trials of anticancer drugs initiated between January 1, 2008, and December 31, 2022

There were 8,364 phase III MRCTs initiated in the US between 1 January 2008 and 31 December 2022. Excluding trials that did not meet the inclusion criteria, there were 999 phase III MRCTs of anticancer agents included in this study (Fig. 1). There was no clear trend in the proportion of cancer trials in the total number of trials.

### Characteristics of the trials

The characteristics of the 999 extracted studies are shown in Table 1. The number of phase III MRCTs increased every 5 years (2008–2012: 239, 2013–2017: 345, and 2018–2022: 415). 35% of the total were minor cancers and 25% were hematological malignancies (all minor cancers). The median number of participating countries was 19 (range: 2–56) and the median sample size was 522. Japan’s participation rate was 51% for the entire period.

**Table 1:** Characteristics of oncology phase III MRCTs started from 2008 to 2022

	*N* = 999
Cancer incidence, *n* (%)	
Major	649 (65)
Minor	350 (35)
Cancer type, n (%)	
Solid tumors	754 (75)
Hematological malignancies	245 (25)
Company size, n (%)	
Large	674 (67)
Non-large	325 (33)
Operational base in Japan, n (%)	
Yes	771 (77)
No	228 (23)
Number of countries, median (range)	19 (2–56)
Sample size, median (range)	522 (0–6000)
Start year	
2008–2012	239 (24)
2013–2017	345 (35)
2018–2022	415 (42)
Japan’s participation, n (%)	
Yes	510 (51)
No	489 (49)
Study design, n (%)	
Comparative	974 (97)
Non-comparative	25 (3)
Primary endpoint, n (%)	
Overall survival	280 (28)
Other	719 (72)

### Japan’s participation in the oncology phase III MRCTs

**Fig. 2:**
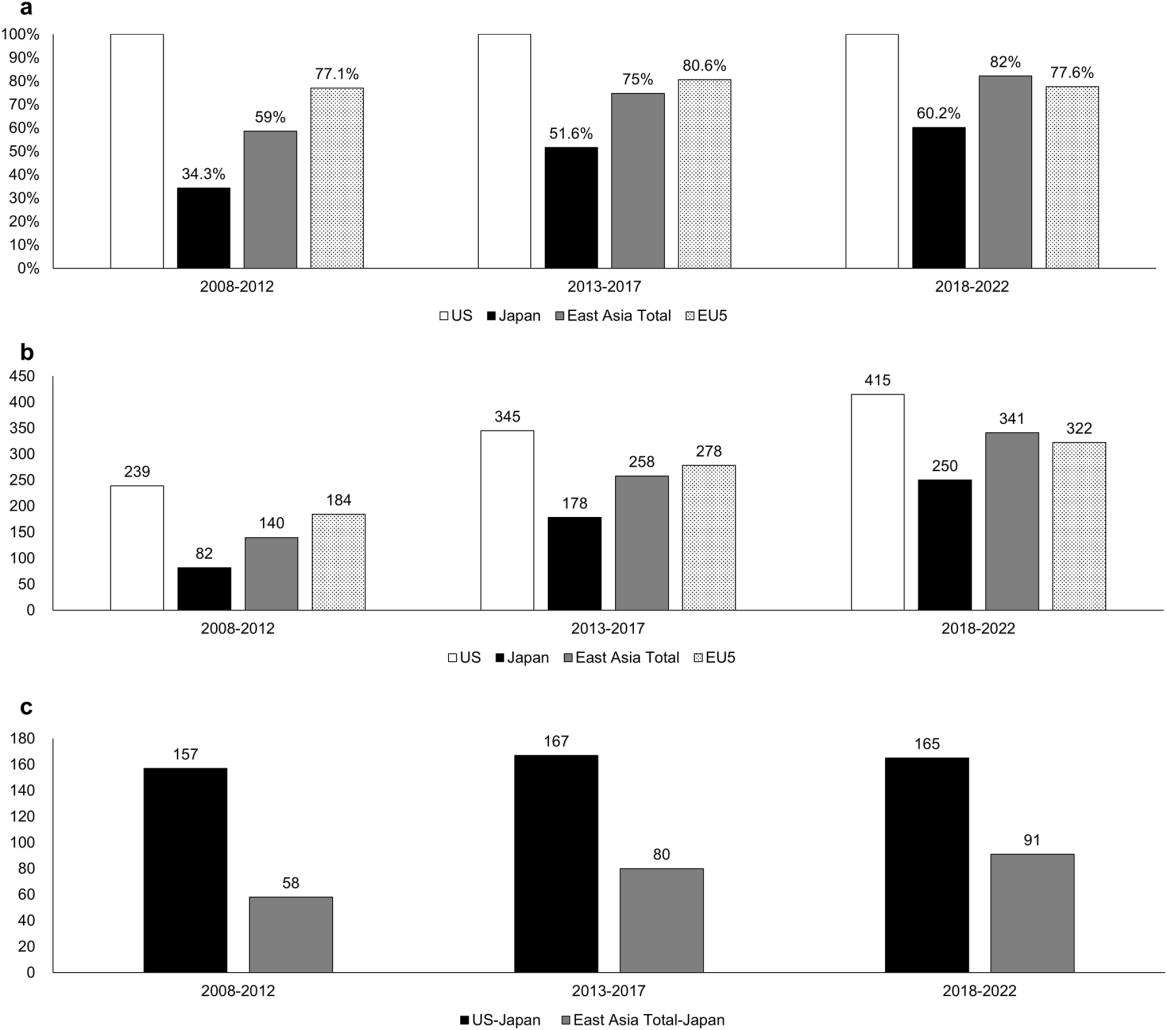
Trends of participation in oncology phase III MRCTs started between 2008 and 2022. **a** Participation rates in oncology phase III MRCTs. **b** Participation numbers in oncology phase III MRCTs. **c** Difference in participation numbers between the US and Japan, and between East Asia Total and Japan

The 5-year trends of the participation rate and participation number in the oncology phase III MRCTs are shown in Fig. 2. As shown in Fig. 2a, the participation rates of the EU5 have remained around 80% over the 15 years, whereas Japan’s participation rates increased from 34 to 60% and East Asia’s total increased from 59 to 82%. However, Japan’s non-participation number has been almost constant (around 160 trials by each 5 years), which resulted in 489 oncology phase III MRCTs that Japan was not included (Fig. 2b). Moreover, among MRCTs that did not include Japan, the number of MRCTs that included East Asian countries other than Japan increased every 5 years to 58, 80, and 91 trials (Fig. 2c).

### Potential factors associated with the Japan’s participation in phase III MRCTs

Table 2 shows the results of the logistic regression analyses for each of the characteristics. In the univariate analysis, non-large sponsor company, sponsor who does not have an operational base in Japan, non-comparative study, major cancer, and hematological malignancies are negative factors for Japan’s participation in oncology phase III MRCTs whereas large trials (whose number of countries are more than 19, whose sample size are more than 522 patients), start year after 2013 (2013–2017 and 2018–2022), and surrogate endpoint are positive factors. In the multivariate logistic regression analysis using variables that were independent among those used in the univariate analysis, sponsor who does not have an operational base in Japan (OR 0.07, 95% CI 0.04–0.11, *p* < 0.01) and minor cancer (OR 0.47, 95% CI 0.34–0.65, *p* < 0.01) were identified as negative factors, and the number of countries is more than 19 (OR 4.00, 95% CI 2.92–5.48, *p* < 0.01), the start year after 2013 (2013–2017: OR 2.49, 95% CI 1.66–3.74, *p* < 0.01; 2018–2022: OR 4.23, 95% CI 2.78–6.43, *p* < 0.01, respectively) were identified as positive factors.

**Table 2:** Results of univariate and multivariate logistic regression analysis

Factors,* p* < 0.05	Univariate logistic regression analysis			Multivariate logistic regression analysis		
	OR	95% CI	*p* value	OR	95% CI	*p* value
Sponsor company profile						
Company size						
Large	Ref	Ref				
Non-large	0.19	0.14–0.25	< 0.01**	–	–	–
Operational base in Japan						
Yes	Ref	Ref				
No	0.06	0.03–0.09	< 0.01**	0.07	0.04–0.11	< 0.01**
Trial profile						
Number of countries						
≤ 19	Ref	Ref				
19 <	5.82	4.43–7.65	< 0.01**	4.00	2.92–5.48	< 0.01**
Sample size						
≤ 522	Ref	Ref				
522 <	2.92	2.26–3.77	< 0.01**	–	–	–
Start year						
2008–2012	Ref	Ref				
2013–2017	2.04	1.45–2.87	< 0.01**	2.49	1.66–3.74	< 0.01**
2018–2022	2.90	2.08–4.04	< 0.01**	4.23	2.78–6.43	< 0.01**
Study design						
Comparative	Ref	Ref				
Non-comparative	0.18	0.06–0.52	< 0.01**			
Primary endpoint						
Overall survival	Ref	Ref				
Surrogate	1.35	1.02–1.78	0.036*	1.04	0.73–1.49	0.827
Cancer incidence						
Major	Ref	Ref				
Minor	0.43	0.33–0.57	< 0.01**	0.47	0.34–0.65	< 0.01**
Cancer type						
Solid tumors	Ref	Ref				
Hematological malignancies	0.65	0.48–0.86	< 0.01**	–	–	–

OR: odds ratio, 95% CI: 95% confidence interval,

*< 0.05, ** < 0.01

### Japan’s participation in the oncology phase III MRCTs by cancer organ

To further investigate the trend of Japan’s participation in oncology phase III MRCTs, we next performed a subgroup analysis by cancer organ. In Table 3, Japan tended to participate in oncology phase III MRCTs when the cancer organs were esophagus (OR 7.78, p = 0.039), head and neck (OR 3.29, *p* < 0.01), lung (R 1.73, *p* < 0.01), stomach (OR 2.71, p = 0.015), and urothelium (OR 2.05, p = 0.036). On the other hand, Japan was unlikely to participate in oncology phase III MRCTs for pancreas (OR 0.15, *p* < 0.01), skin (OR 0.23, *p* < 0.01), and soft tissue (OR 0.36, *p* = 0.013). Among hematological malignancies, Japan was unlikely to participate in leukemia trials (OR 0.40, *p* < 0.01), while there was no such trend in terms of lymphoma and myeloma.

**Table 3:** Japan’s participation in oncology phase III MRCTs by cancer organ

Cancer organ	Cancer type	Total	With Japan, *n* (%)	OR	95% CI	*p* value
Biliary tract	Solid tumors	4	4 (100)	∞	∞	0.125
Brain	Solid tumors	12	4 (33)	0.48	0.14–1.59	0.255
Breast	Solid tumors	116	67 (58)	1.36	0.92–2.01	0.139
Cervix	Solid tumors	7	5 (71)	2.41	0.47–12.49	0.452
Colorectum	Solid tumors	27	18 (67)	1.95	0.87–4.39	0.119
Esophagus	Solid tumors	9	8 (89)	7.78	0.97–62.41	0.039*
Head and neck	Solid tumors	26	20 (77)	3.29	1.31–8.25	< 0.01**
Kidney	Solid tumors	34	19 (56)	1.22	0.61–2.43	0.604
Leukemia	Hematological malignancies	120	38 (32)	0.40	0.27–0.60	< 0.01**
Liver	Solid tumors	36	24 (67)	1.96	0.97–3.97	0.063
Lung	Solid tumors	177	110 (62)	1.73	1.24–2.42	< 0.01**
Lymphoma	Hematological malignancies	68	32 (47)	0.84	0.51–1.38	0.531
Mesothelium	Solid tumors	6	2 (33)	0.48	0.09–2.62	0.443
Myeloma	Hematological malignancies	57	35 (61)	1.56	0.90–2.70	0.133
Neuroendocrine	Solid tumors	7	1 (14)	0.16	0.02–1.32	0.064
Ovary	Solid tumors	39	16 (41)	0.66	0.34–1.26	0.253
Pancreas	Solid tumors	22	3 (14)	0.15	0.04–0.50	< 0.01**
Prostate	Solid tumors	69	29 (42)	0.68	0.41–1.11	0.135
Skin	Solid tumors	45	9 (20)	0.23	0.11–0.47	< 0.01**
Soft tissue	Solid tumors	29	8 (28)	0.36	0.16–0.81	0.013*
Stomach	Solid tumors	30	22 (73)	2.71	1.20–6.15	0.015*
Thyroid	Solid tumors	8	4 (50)	0.96	0.24–3.85	1.000
Urothelium	Solid tumors	40	27 (68)	2.05	1.04–4.01	0.036*
Uterus	Solid tumors	9	4 (44)	0.77	0.20–2.87	0.748

OR: odds ratio, 95% CI: 95% confidence interval,

*< 0.05, **< 0.01

### Japan’s participation in the oncology phase III MRCTs by drug modality

In terms of drug modalities, Japan is likely to participate in antibody trials (especially monoclonal antibodies: OR 3.03, *p* < 0.01) and unlikely to participate in trials of protein/peptide (OR 0.18, *p* < 0.01), nucleic acid (OR 0.00, *p* = 0.028), small molecule (especially cytotoxic agents: OR 0.09, *p* < 0.01), and radiotherapies (OR 0.13, *p* < 0.01). On contrast, there was no trend in new modalities such as cell therapies (e.g., CAR-T), gene therapies and other new modality trials.

## Discussion

This study aimed to investigate the situation of “potential drug lag/drug loss” based on Japan’s participation rate in phase III MRCTs of anticancer drugs to predict future drug lag and drug loss.

The data from our study indicate that for anticancer drugs, Japan’s participation rate in phase III MRCTs has increased over the past 15 years, while the number of non-participations has not decreased, which potentially indicates there might still be drug approval lag or even drug loss in the future. This suggests that the efforts taken by the Japanese government, regulatory authorities, and industry were at least partially successful in terms of Japan’s participation rate in MRCTs. On the other hand, in terms of unapproved drugs, there could be space for making efforts to improve. Previous reports indicate that the drug lag in the field of rare diseases is increasing when unapproved drugs are taken into account, and that many unapproved drugs are being developed by non-Japanese companies without Japan’s participation in global clinical trials [11]. Our study differs from the previous report in that the analysis was based on phase III MRCTs of anticancer drugs, but the results suggest that the presence or absence of an operating base in Japan affects the participation rate of Japan in oncology phase III MRCTs, which is consistent with the trend reported in the previous report [11].

In addition, a previous report, which analyzed US-approved cancer drugs by drawing a Kaplan–Meier curve with unapproved drugs treated as “censored” and Japanese approval as the event, reported a median approval lag of 961 days for 2011–2016 and 1555 days for 2017–2022, indicating that the approval lag is increasing, mainly due to an increase in unapproved drugs [21]. Our study demonstrated that the approval lag is expected to expand, and the number of unapproved drugs will continue to increase in the future as well because the number of non-participations of Japan in phase III MRCTs has not decreased. Interestingly, the number of cases in which Japan did not participate but other East Asian countries (Korea, China, Hong Kong, or Taiwan) have participated increased over the past 15 years. The introduction of ICH E17 [22] may have lowered Japan’s priority for inclusion in the MRCTs when considering East Asia as a region. East Asian countries other than Japan have large hospitals with many beds; therefore, they can enroll many patients with a small number of sites in MRCTs. On the other hand, since Japan does not have large hospitals like other countries, more sites are needed for patient enrollment, resulting in higher costs. The gap between East Asia and Japan is narrowing for major cancers (Fig. 4b), while the gap between East Asia and Japan is widening for minor cancers (Fig. 4d), suggesting that Japan’s position is declining especially in minor cancers. In the case of minor cancers, it is necessary to use rare cancer registries such as MASTER Key [23] for accelerated patient enrollment.

Since non-large companies that do not have an operating base in Japan are less likely to participate in MRCTs in Japan (Fig. 3b), it is necessary to promote drug development in Japan by these companies (many of which are overseas start-up companies). Reasons why small overseas companies do not develop drugs in Japan may be 1) the Japanese drug price system, which does not allow pharmaceutical companies to freely set drug prices and offers constantly decreasing drug prices, 2) the cost performance of conducting clinical trials in Japan is poor, and 3) licensing and In-Country Clinical Care-taker (ICCC) clinical trials’ implementation is time-consuming and labor-intensive [24]. Resolution of these issues is needed. Recently, the MHLW has clarified the need for Japanese data in the regulatory review process, and in principle, additional Japan phase I studies are not required when participating in global clinical trials [25]. Although it is said for anticancer drugs that, from the viewpoint of safety, more careful judgment is needed to determine whether Japan phase I studies are required or not, the MRCT participation rate for anticancer agents in Japan is expected to also increase in the future. In this study, there were differences in Japan’s participation/non-participation in MRCTs according to cancer incidence, and the likelihood of Japan’s participation in MRCTs was low for minor cancers. The main reason for this could be the increasing number of applications and approvals based on early stage clinical trials for minor cancers. The approval lag between Japan and the US for major cancers improved from 2006–2011 to 2012–2016, but there was no improvement for minor cancers, and a development strategy using MRCTs is reportedly needed to shorten the approval lag [18]. In terms of regulatory characteristics, Breakthrough Therapy Designation by FDA and/or Accelerated Approval is associated with a shorter approval lag in Japan [26], and in the US, Breakthrough Therapy Designation and/or Accelerated Approval is more common in minor cancers [27]. Pivotal trials for orphan anticancer drugs tend to be early-stage, non-randomized studies whose primary endpoint is response rates [28]. To decrease the drug lag in rare cancers, it is necessary to promote Japan’s participation not only in phase III MRCTs but also in early phase MRCTs. Since the trend of Japan’s participation in oncology phase I MRCTs has differed depending on the sponsor company as reported in a previous report [29], action to include Japan in phase I MRCTs may be necessary, especially for companies that have a low trend of Japan’s participation in phase I MRCTs.

Interestingly, Japan’s participation in MRCTs varied by target cancer organ. Japan’s participation in MRCTs tended to be high for lung and stomach cancers, which are the most common site-specific cancers among Japanese, suggesting that drug development is driven by medical needs. On the other hand, Japan’s participation in MRCTs for pancreatic cancer, which is one of the main causes of cancer death among Japanese, tended to be low. And the number of MRCTs themselves was also low. This indicates the unmet medical need in pancreatic cancer and the need for further improvement in Japan.

Also, differences in Japan’s participation rate in MRCT were observed by drug modality. The number of small molecule and monoclonal antibody trials for molecular targets has increased since the MHLW/PMDA measures to promote international joint clinical trials, and Japan’s participation rate in phase III MRCT has been considered high because many of them were conducted by companies that have operational bases in Japan (Table 4), while Japan’s participation in phase III MRCT is low when it comes to cytotoxic agents because many cytotoxic agents have been approved through bridging or public knowledge-based application in Japan [30]. In fact, in the MRCT analysis of all diseases, the odds ratio (OR) for Japan’s participation in MRCT was 2.19 (1.33–3.62, *p* = 0.002) for biological drugs, while the OR for chemical compounds was 0.63 (0.39–0.99), a trend similar to that of the present study [31]. In this study, the phase III MRCTs for protein/peptide, cell therapies, gene therapies, nucleic acids, and radiotherapies tended to be led by companies that did not have operational bases in Japan, which may explain the low participation rate of MRCTs in Japan (Table 4). These issues highlight the challenges Japan faces in drug development for new modalities.

**Table 4:** Japan’s participation in oncology phase III MRCTs by drug modality

Drug modality	Total	With Japan, *n* (%)	OR	95% CI	*p* value
Antibodies	391	267 (68)	3.23	2.47–4.22	< 0.01**
Monoclonal antibodies	318	220 (69)	3.03	2.28–4.01	< 0.01**
Antibody–drug conjugates	57	36 (63)	1.69	0.97–2.94	0.075
Bispecific antibodies	16	11 (69)	2.13	0.74–6.19	0.208
Proteins and peptides	64	11 (17)	0.18	0.09–0.35	< 0.01**
Cell therapies	14	5 (36)	0.53	0.18–1.59	0.290
Gene therapies	3	1 (33)	0.48	0.04–5.29	0.617
Nucleic acids	5	0 (0)	0.00	0.00–0.00	0.028*
Small molecule	494	221 (45)	0.61	0.47–0.78	< 0.01**
Cytotoxic agents	53	5 (9)	0.09	0.04–0.23	< 0.01**
Molecular-targeted drugs	441	216 (49)	0.86	0.67–1.10	0.252
Radiotherapies	16	2 (13)	0.13	0.03–0.59	< 0.01**
Other new modalities	12	3 (25)	0.32	0.08–1.17	0.085

OR: odds ratio, 95% CI: 95% confidence interval,

*< 0.05, **< 0.01

There are limitations in our study. Including only phase III trials as a target is a limitation since the number of approvals based on early stage trials such as phase I and II studies as pivotal trials has increased in Japan for mainly rare cancers in recent years. In the U.S., the FDA often grants accelerated approval based on early stage trials and then grants full approval with the results of the phase III trial as a confirmation trial. In Japan, SAKIGAKE designation and conditional early approval for new drug applications are equivalent to the FDA’s Breakthrough Therapy designation and Accelerated Approval [32, 33], however, there are not yet many examples of use, and it is hoped that the early approval system will be expanded to the same level as that of the FDA.

Many previous reports have analyzed the drug lag resulting from drug approval dates in the US and Japan [8, 12, 18, 26]. On the other hand, this study is significant in that it can predict the possibility of a lag not only in past trends but also in drugs/indications scheduled for approval in the future, based on the inclusion rate of Japan in phase III MRCTs.

The number of Japan’s non-participation in phase III MRCTs continues to increase, and future expansion of drug lag and drug loss was anticipated. Promotion of Japan’s inclusion in the MRCTs and expansion of the early approval system are desirable.

## Supplementary Information

Below is the link to the electronic supplementary material.

Supplementary file1 (TIF 443 KB)

Supplementary file2 (TIF 415 KB)

Supplementary file3 (TIF 220 KB)

Supplementary file4 (TIF 245 KB)

Supplementary file5 (TIF 219 KB)

Supplementary file6 (TIF 241 KB)
