# Supplementary figures and images for: Evaluation of drug lag and drug loss in Japan: participation in global phase III oncology trials
Source: Int J Clin Oncol. 2025 Apr 11;30(6):1109–17. doi: 10.1007/s10147-025-02756-8 (PMC12122644; doi:10.1007/s10147-025-02756-8)

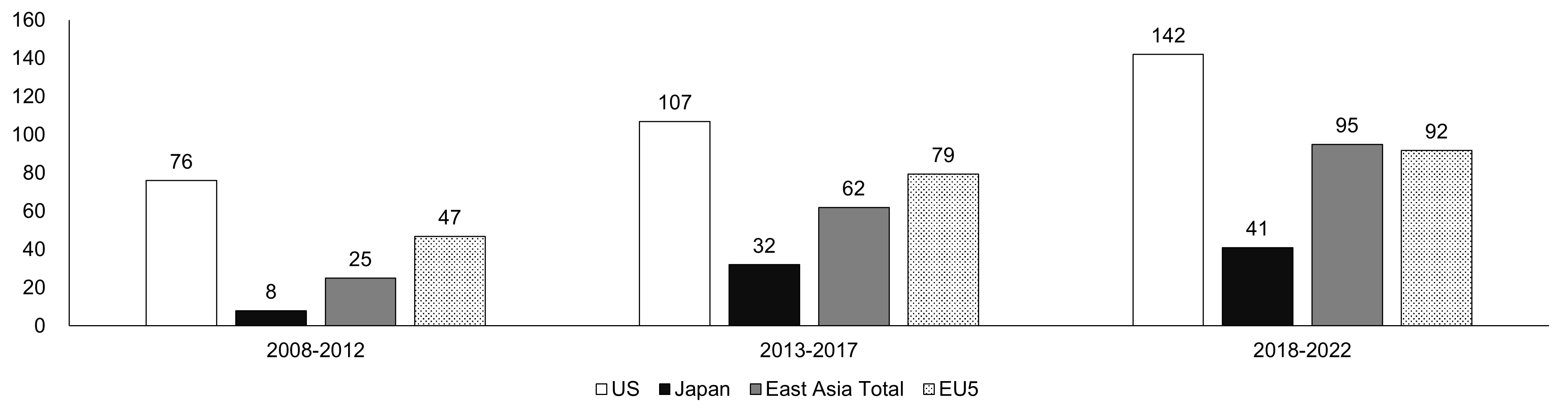

Supplement: Supplementary file 1 — Supplementary file1 (TIF 443 KB) [file 10147_2025_2756_MOESM1_ESM.tif]

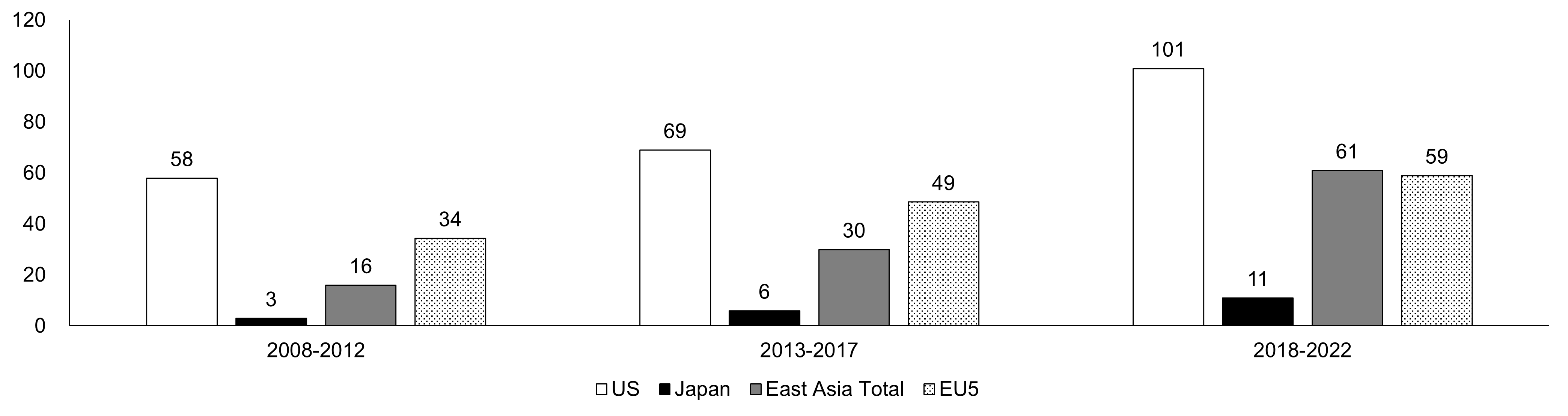

Supplement: Supplementary file 2 — Supplementary file2 (TIF 415 KB) [file 10147_2025_2756_MOESM2_ESM.tif]

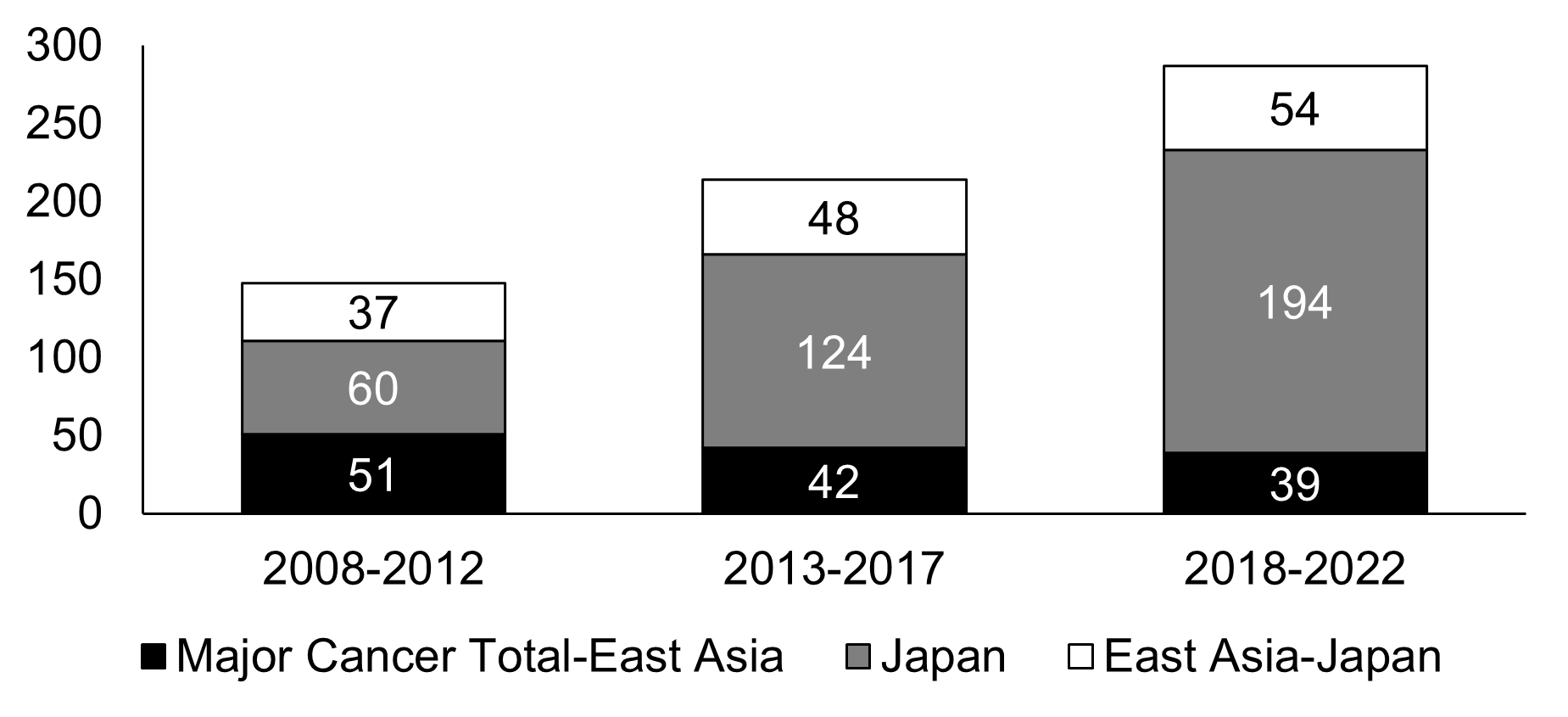

Supplement: Supplementary file 3 — Supplementary file3 (TIF 220 KB) [file 10147_2025_2756_MOESM3_ESM.tif]

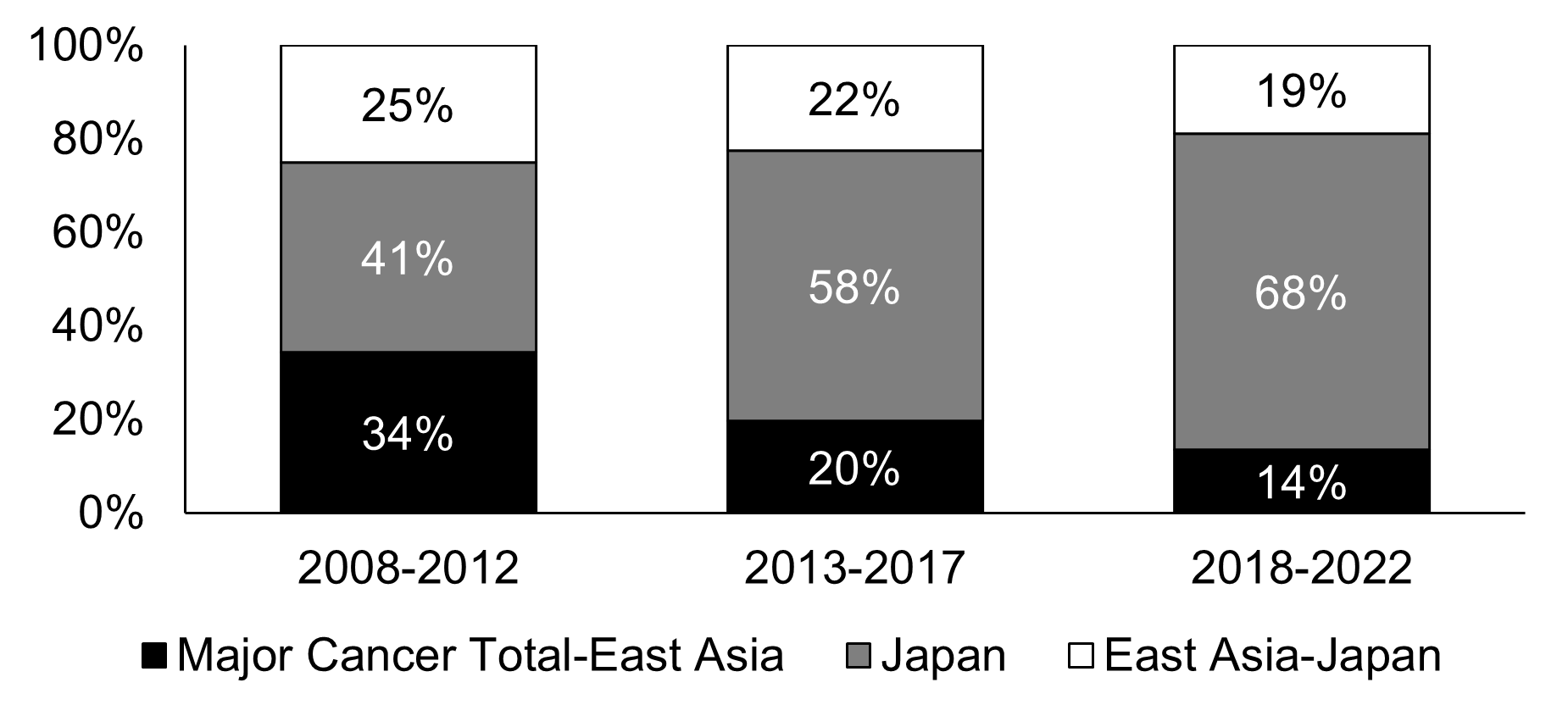

Supplement: Supplementary file 4 — Supplementary file4 (TIF 245 KB) [file 10147_2025_2756_MOESM4_ESM.tif]

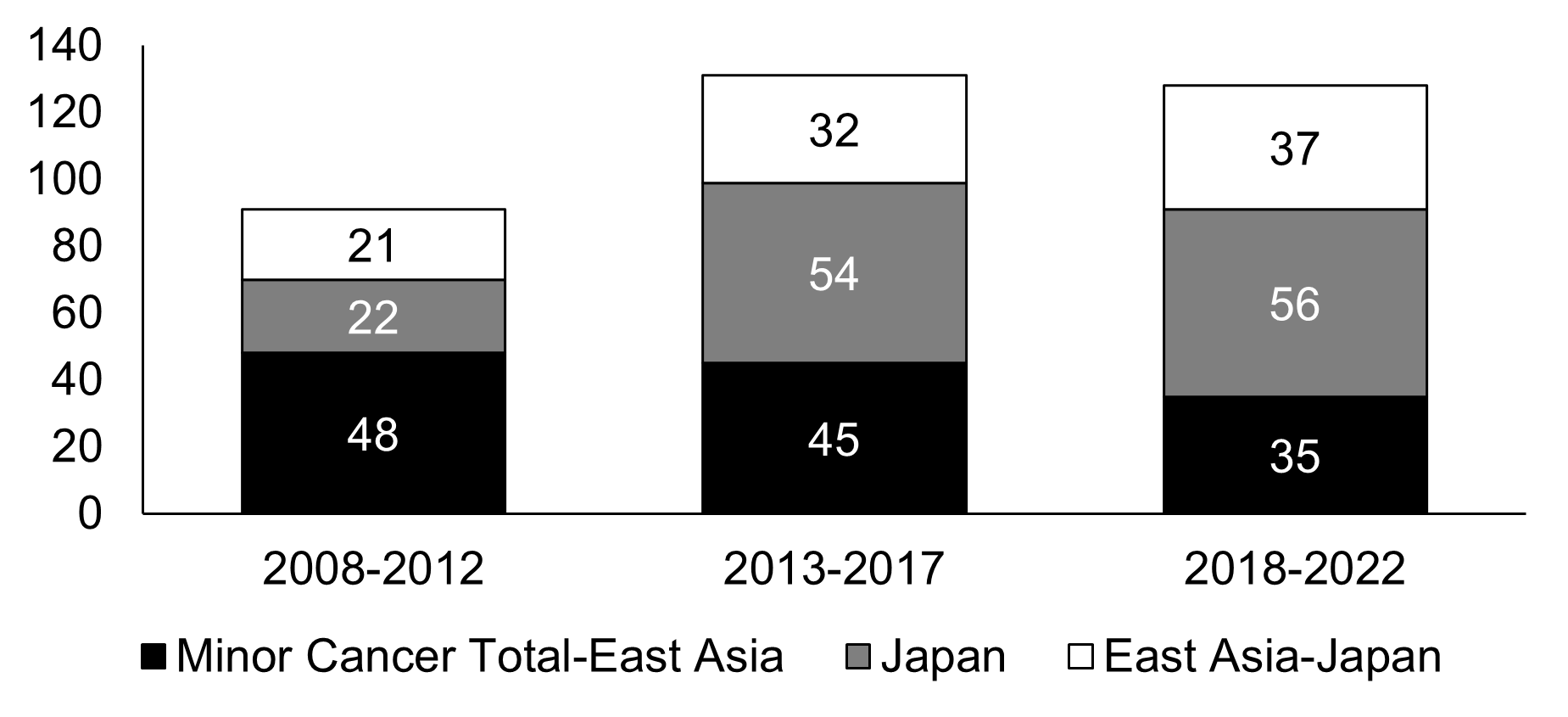

Supplement: Supplementary file 5 — Supplementary file5 (TIF 219 KB) [file 10147_2025_2756_MOESM5_ESM.tif]

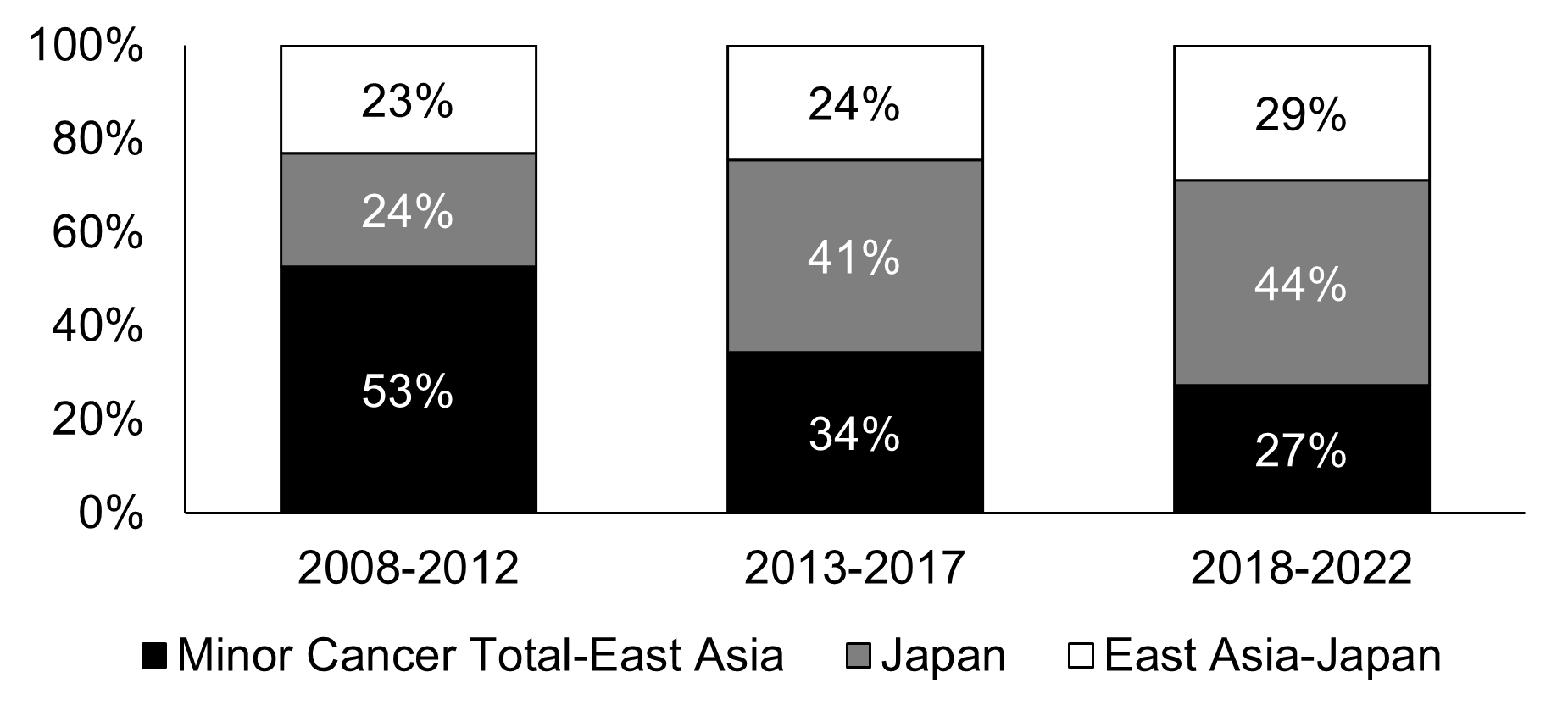

Supplement: Supplementary file 6 — Supplementary file6 (TIF 241 KB) [file 10147_2025_2756_MOESM6_ESM.tif]
